# D3, the new diffractometer for the macromolecular crystallography beamlines of the Swiss Light Source

**DOI:** 10.1107/S160057751400006X

**Published:** 2014-02-04

**Authors:** Martin R. Fuchs, Claude Pradervand, Vincent Thominet, Roman Schneider, Ezequiel Panepucci, Marcel Grunder, Jose Gabadinho, Florian S. N. Dworkowski, Takashi Tomizaki, Jörg Schneider, Aline Mayer, Adrian Curtin, Vincent Olieric, Uli Frommherz, Goran Kotrle, Jörg Welte, Xinyu Wang, Stephan Maag, Clemens Schulze-Briese, Meitian Wang

**Affiliations:** a Swiss Light Source, Paul Scherrer Institute, 5232 Villigen PSI, Switzerland; b NSLS-II, Brookhaven National Laboratory, Mail Stop 745, Upton, NY 11973, USA; c DECTRIS Ltd, Neuenhoferstrasse 107, 5400 Baden, Switzerland

**Keywords:** macromolecular crystallography, diffractometer, microspectrophotometer, microcrystallography, beamline endstation

## Abstract

A new diffractometer for microcrystallography has been developed for the three macromolecular crystallography beamlines of the Swiss Light Source. Building upon and critically extending previous developments realised for the high-resolution endstations of the two undulator beamlines X06SA and X10SA, as well as the super-bend dipole beamline X06DA, the new diffractometer was designed to the following core design goals. (i) Redesign of the goniometer to a sub-micrometer peak-to-peak cylinder of confusion for the horizontal single axis. Crystal sizes down to at least 5 µm and advanced sample-rastering and scanning modes are supported. In addition, it can accommodate the new multi-axis goniometer PRIGo (Parallel Robotics Inspired Goniometer). (ii) A rapid-change beam-shaping element system with aperture sizes down to a minimum of 10 µm for microcrystallography measurements. (iii) Integration of the on-axis microspectrophotometer MS3 for microscopic sample imaging with 1 µm image resolution. Its multi-mode optical spectroscopy module is always online and supports *in situ* UV/Vis absorption, fluorescence and Raman spectroscopy. (iv) High stability of the sample environment by a mineral cast support construction and by close containment of the cryo-stream. Further features are the support for *in situ* crystallization plate screening and a minimal achievable detector distance of 120 mm for the Pilatus 6M, 2M and the macromolecular crystallography group’s planned future area detector Eiger 16M.

## Abstract 2

A new diffractometer for microcrystallography has been developed for the three macromolecular crystallography beamlines of the Swiss Light Source.

## 1. Introduction   

Diffractometer endstations at synchrotron beamlines for macromolecular crystallography (MX) see a multitude of different users; they come from university research groups, large-scale biomolecular research institutes and the pharmaceutical industry. As much as the users vary, so do the use cases: from the *de novo* structure determination of large biomolecular complexes, over high-throughput screening of drug targets, room-temperature crystal screening and functional studies, to the collection of complete datasets from ever smaller crystals or sets of crystals down to a few micrometers in size.

This breadth of requirements can be covered either by developing specialized equipment and rebuilding between measurements, or by designing a highly flexible multi-configuration instrument. An example of a very successful highly integrated generic macromolecular crystallography (MX) diffractometer is the microdiffractometer MD2 developed by the EMBL Grenoble (Perrakis *et al.*, 1999 ▶), which was originally developed for the ESRF/EMBL MX beamlines (eight installations), but has since been commercialized (MAATEL, Bruker EST) and installed at MX beamlines in at least ten synchrotrons worldwide. Other beamlines, such as, for example, the GM/CA beamlines at sector 23 of the APS (Xu *et al.*, 2010 ▶), share a mostly common home-built diffractometer design between different endstations. Often, the choice of a central component such as the goniometer can dominate the design and layout of the diffractometer, such as, for example, the Kappa multi-axis goniometer (Crystal Logic) of the SOLEIL PROXIMA1 beamline (Ascone *et al.*, 2007 ▶; Goulet *et al.*, 2010 ▶). An extreme example of a highly configurable endstation design is the instrument currently being commissioned at the PETRA III MX and biological imaging beamline P11 (Fischer *et al.*, 2012 ▶), which will integrate separate instruments for MX, imaging and time-resolved measurements. The approach adopted in the design of the new ‘D3’ diffractometer for the Swiss Light Source (SLS) MX beamlines was to develop a system that is generic where possible, while still allowing for beamline- or application-specific specialized developments to be easily included in the set-up where necessary.

At the Paul Scherrer Institute (PSI), the SLS MX group operates three synchrotron beamlines dedicated to MX, and with their configuration caters to all the user communities mentioned above. These three beamlines together have produced more than 2900 macromolecular structures over the last decade (BioSync, 2013 ▶). Two of the beamlines, X06SA (Schulze-Briese *et al.*, 2001 ▶) and X10SA (Pohl *et al.*, 2006 ▶), are undulator beamlines, while the third and youngest beamline, X06DA (Diez *et al.*, 2007 ▶), is fed by a super-bend dipole magnet. These beamlines were opened for user operation over a period of seven years. In 2001 the X06SA/PXI high-resolution endstation was inaugurated. In 2005 X10SA/PXII started operation with a design essentially a copy of the successful X06SA layout, and in 2008 the mini-hutch design of the X06DA/PXIII beamline was finalized. All of the endstations of the beamlines received numerous specialized upgrades over the years; for example, the addition of a separate microfocus endstation at X06SA in 2004, an on-axis microscope and beam-shaping apertures with an optional microspectro­photometer at X10SA (Pompidor *et al.*, 2013 ▶), and an *in situ* plate-screening facility at X06DA (Bingel-Erlenmeyer *et al.*, 2011 ▶).

While this specialization provided the user community with powerful new data acquisition methods, it also brought about a considerable diversification of the experimental set-ups. The new diffractometer project was initiated with the intention to make the best developments of the separate beamlines available at all MX beamlines at the SLS, as well as to standardize the endstations as much as possible. One example for such ‘best developments’ is the goniometer set-up of X06DA, where the horizontal air-bearing rotation table sits on a full *XYZ* translation stack, enabling rapid refocusing of the sample-viewing optics by simply moving the sample into the microscope focus. This increases the microscope stability by removing the need of a variable focusing optics. Another example is the retractable beam-shaping apertures at beamline X10SA, which enabled the use of a microbeam without the requirement for an extra X-ray focusing stage.

The upgrade also offered the opportunity to improve key performance figures such as the cylinder of confusion of the goniometer, with a goal of ≤1 µm peak-to-peak, and the minimal detector-to-sample distance, with a goal of ≤120 mm. Further, the goniometer and sample translation stages were specified to meet the precision and reproducibility requirements for advanced sample centering and data acquisition schemes such as raster scanning over a grid and continuous scanning along a path, also called ‘helical scanning’ (Song *et al.*, 2007 ▶; Cherezov *et al.*, 2009 ▶; Aishima *et al.*, 2010 ▶; Flot *et al.*, 2010 ▶).

The improvement of the diffractometer performance clearly should go along with an increase in instrument uptime and reliability, as well as ease of use. Therefore, the reliability and reproducibility of all components had to be optimized, with a special focus on stability by reducing thermal drifts due to the sample cooling and the sample changer operation, as well as isolation from vibrations. Careful integration of all components was required to both make optimal use of the limited sample space and also to facilitate the collision avoidance of the moveable components. Last but not least, quick access to all components for maintenance had to be ensured.

The advantages of a standardization of central components across all three beamlines are manifold. Firstly, future optimizations of hardware and software will easily carry over to the other beamlines. Secondly, the redundancy of critical spare parts can be drastically reduced. Thirdly, by offering users and the beamline staff a unified interface at all the beamlines, both the efficient use of the beamline resources as well as the development of new components are improved. Lastly, by implementing a dummy set-up in an adjacent offline laboratory, quick testing of hardware and software for all three beamlines becomes possible.

The installation was carried out within three weeks during a regular synchrotron shutdown in April 2012. To ensure successful commissioning and full user operation immediately after the shutdown, the complete diffractometer was pre-assembled at the MX group’s technical laboratories, where all critical components underwent site acceptance tests. The instrument was then partially dismounted for transporting to beamline X10SA for the final installation and full user operation could be resumed. Since September 2012, the full functionality of the diffractometer was made available to the users, including a mini-beam rastering mode and concurrent spectroscopy experiments. Its design principles and realisation will be presented in this paper.

## 2. Hardware   

The general layout of the diffractometer arranges most components on a common base, the D3 table. The remaining devices are attached from above to the A-frame detector support (Rosenbaum *et al.*, 2006 ▶). The design thus could be kept as modular as possible, to both vibrationally isolate components from each other as well as maintain a high degree of configurability. Some details of the PSI construction and engineering have been described by Maag *et al.* (2013 ▶).

### 2.1. Goniometer   

**Figure 1:**
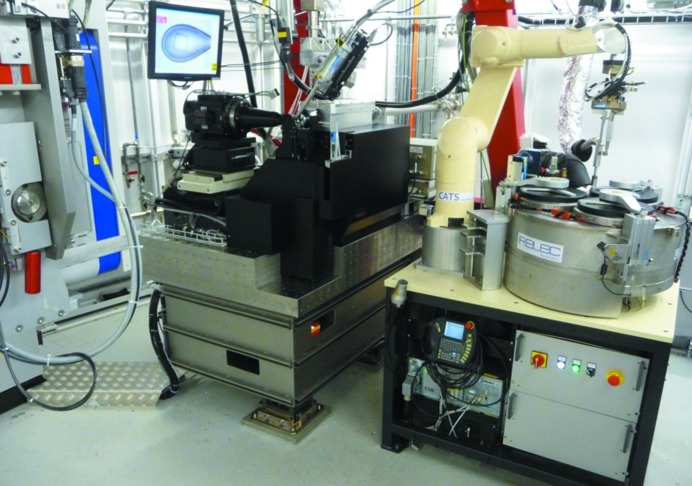
Experimental endstation of beamline X10SA. From left to right: Pilatus 6M area detector (Dectris), D3 diffractometer, CATS robotic sample changer system (IRELEC).

The new goniometer (Fig. 1 ▶) has to meet several critical specifications requirements. To support the data acquisition from crystals down to a size of at least 5 µm, the cylinder of confusion needs to be below 1 µm peak-to-peak. For a crystal size matching the vertical beam size of 10 µm this corresponds to a criterion of deviations of the overlap to be <10% and for smaller crystal sizes the crystal should remain centered at the beam maximum to minimize scattering from the surrounding buffer. Further, for scanning and rastering data acquisition modes, the positional reproducibility for translations in all spatial directions needs to be below 1 µm and a synchronization of the stages’ movement with the data acquisition system (shutter and detector) must be provided by position synchronized output (PSO). In addition to this, the gonio­meter needs to support the installation of the PRIGo multi-axis goniometer (Glettig *et al.*, 2011 ▶), leading to a requirement of a minimum load capacity of 4 kg and a sample center distance to the rotation table of 300 mm. A further requirement is the compatibility with the existing controls environment employing an EPICS (EPICS, 2012 ▶) IOC interfaced to an Aerotech A3200 controller.

**Figure 2:**
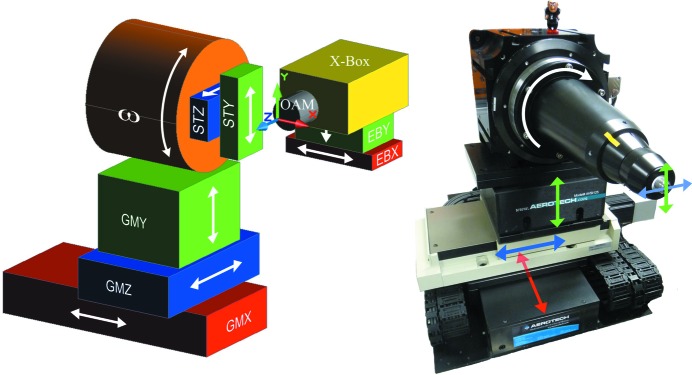
Left: degrees of freedom (DOF) of the D3 diffractometer. The translations GMX, STY and STZ and the rotation ω are used for scanning operations, *i.e.* with the X-ray shutter opened, the translations GMY and GMZ are used for sample positioning during alignment only. The active X-ray beam feedback to an X-ray beam-position monitor in the so-called exposure box (X-Box) allows the positioning of the beam *via* the EBX and EBY translations. The on-axis microscope is the reference device to which all others are aligned; the sole DOF with a relation to the microscope is the GMZ translation of the goniometer that is used to bring the sample into focus. Right: DOF mapped onto the actual goniometer.

The goniometer consists of an air-bearing rotation axis for the ω rotation (Aerotech ABRT 200) mounted on top of a stack of linear translation stages (see Fig. 2 ▶, which also contains the definition of the coordinate axes). All cable connections to positioners mounted on the rotation table are fed through a slip ring (Moog AC6355) with 56 connections to provide sufficient contacts for the later integration of the PRIGo goniometer. To reduce the contribution of the slip ring to the tilt error motion, a second direct-drive rotary table with mechanical bearings (Aerotech ADRT-150-115) is implemented as a slip ring carrier and moves the slip ring co-axially to and synchronously with ω. The translations are, from bottom to top: GMX, parallel to the rotation axis (Aerotech ALS25020), GMZ, parallel to the X-ray beam (Aerotech ATS20010) and GMY, vertical (Aerotech AVSI125). The GMX stage is a direct-drive linear stage; for sample centering and scanning operations with open shutter, the GMY and GMZ stages are a ball-screw lift stage and linear stage, respectively, to position the rotation axis at the height of the beam and in the focus of the on-axis microscope. Two sample-centering stages for the *Y*- and *Z*- directions (SmarAct SLC-1720-S), STY and STZ, are mounted on the table of the ω axis, directly supporting the sample magnet. The sample centering stages are closed-loop piezo positioners with a 4 mm travel range. Initially the centering stages were driven independently from the Aerotech control system *via* an in-house-developed driver, the SLS 3603 PMD. Their control has now been integrated into the goniometer A3200 controller *via* an Aerotech Nstep-4 stepper motor hardware interface, thus enabling real time synchronized control of all goniometer degrees of freedom. This combination of all goniometer actuators into one control system considerably facilitates the implementation of advanced data acquisition schemes as well as the integration of calibration-based corrections of reproducible stage error motions.

**Figure 3:**
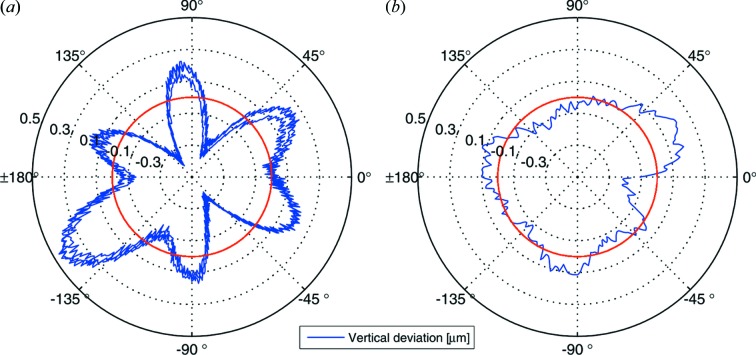
Vertical deviations of the sample-mounting position during rotations of the stage. The plot shows the integral error contributions of the horizontal air-bearing stage and the sample centering and support mounted on top of the rotation table. (*a*) Uncorrected error during three successive rotations. (*b*) Calibration-corrected signal. The average position is marked by the red circle.

For the site acceptance tests of the goniometer system, different types of measurements were performed. To determine the integral error motion of the system at the sample position, capacitive distance sensors (LION Precision C23-B, resolution < 2 nm RMS, range 50 µm) were used (Salathe, 2010 ▶) to verify the sub-micrometer positional reproducibility of the sample position upon movement of the goniometer translation stages. Furthermore, the vertical and horizontal deviation of a metal precision sphere mounted on a SPINE sample cap was measured for rotations around ω as a measure for the sample cylinder of confusion (a figure of the set-up is provided in the supporting information^1^). As the metal spheres (diameter 12.7 mm) weigh considerably more than the crystal mounting pins, the measured deviations are a conservative upper limit for the real deviations of the rotation axis and thereby the sample mounting position during crystallographic data acquisition. Measured in this way, the vertical deviation was determined to be 880 nm peak-to-peak with a standard deviation of 190 nm (Fig. 3*a* ▶). The error motion is largely reproducible, as is demonstrated by the overlay of the curves of three successive full rotations, with the irreproducible error band being well below 200 nm. The symmetry of the deviation curve can serve as a hint to the origin of the deviations, with the sixfold pattern possibly correlated with the air-bearing spindle construction and the less prominent twofold pattern possibly correlated with the centering stages, with both devices still performing well within their specifications.

Since the largest part of the error amplitude is reproducible, by tabulating the average error as a function of the rotation angle and applying an appropriate correction to the sample position with the STY and STZ sample centering stages the cylinder of confusion can be minimized. In a first proof-of-principle test, by applying a centering correction every 2°, a corrected vertical deviation of 425 nm peak-to-peak and a standard deviation of 90 nm was obtained (Fig. 3*b* ▶). This shows that the mechanical properties of the goniometer provide significant potential for improvement beyond the boundary conditions set for the current beam sizes and for accommodation of X-ray optics upgrades to smaller beam sizes. The systematic error due to the imperfect shape of the measurement sphere was eliminated with the Donaldson ball reversal method (Donaldson, 1972 ▶). Once the control of the sample centering stages is integrated into the Aerotech EPICS IOC, the correction look-up table can be applied synchronously to the stages motion and this calibration correction will be applied permanently.

In addition, critical subunits of the goniometer assembly were commissioned separately. The tilt errors specifications of the air-bearing rotation axis (≤ 3 µrad peak-to-peak) were verified in measurements with an autocollimator (Möller-Wedel Elcomat 2000) and a flat mirror mounted on the rotation table. Similarly, the flatness and straightness specifications of the linear stages critical for scanning operation were determined with measurements with a laser interferometer (HP 5526A).

As the diffractometer was developed to fit all three MX beamlines, an identical goniometer was built simultaneously for the second undulator beamline X06SA and commissioned. As for the first system, a sub-micrometer cylinder of confusion was obtained (data not shown). For the bending-magnet beamline X06DA, due to its larger vertical beam size of 45 µm, the current goniometer system with the PRIGo multi-axis goniometer cylinder of confusion of <3 µm peak-to-peak will be kept (final specifications to be published).

### 2.2. Sample environment   

To ensure that the PRIGo multi-axis goniometer (Glettig *et al.*, 2011 ▶) can be integrated into the diffractometer set-up without any further modifications of the diffractometer, extensive integration studies were carried out during the construction phase of the single-axis system (Maag *et al.*, 2013 ▶). In these studies, potential collisions with the lamp, the microscope, the beam-shaping devices and the cryogenic system were investigated. For the final design of the PRIGo, minor required modifications to the positioning of the χ-axis and its supporting tripod were identified, with which the integration into the D3 diffractometer can be achieved.

**Figure 4:**
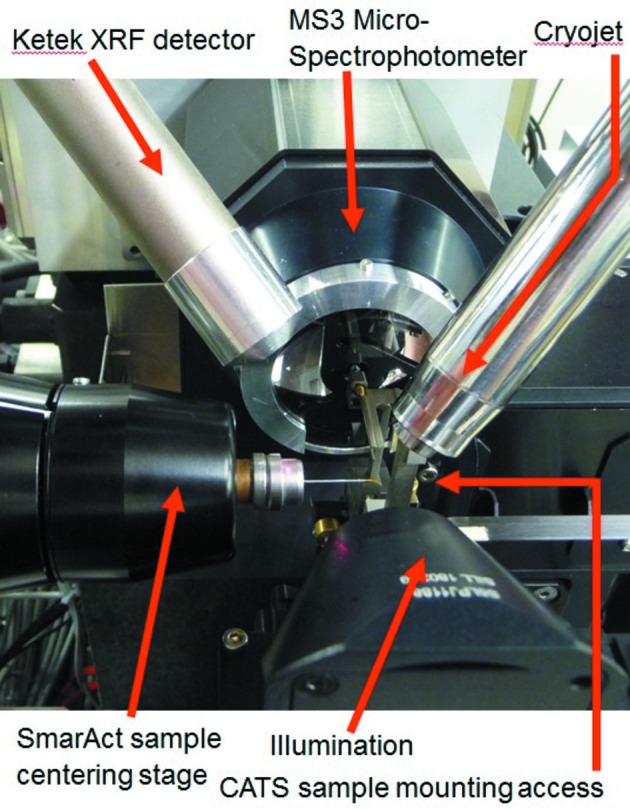
Sample environment of the D3 with all devices positioned in closest sample proximity.

**Figure 5:**
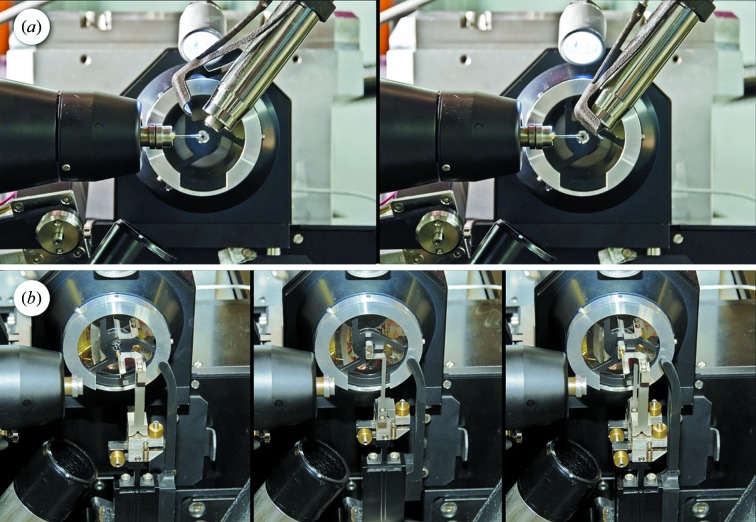
(*a*) Cryo-stream shutter for remotely controlled sample annealing with a pneumatically actuated flexor mechanism. The unit is fabricated in a rapid prototyping polyamide process based on the design by Helmholtz Zentrum Berlin (Mueller *et al.*, 2012 ▶). Left: open position, default. Right: blocking position. The cold stream is diverted to the bottom right direction in the image. (*b*) Beam-shaping devices, downstream of the reflective objective of the microspectrophotometer. Left: scatter guard collimator, a Mo tube with 0.5 mm outer and 0.3 mm inner diameter, to confine air scatter from the beam up to 5 mm upstream of the sample. Center: triple aperture. Right: data collection configuration with aperture and collimator in the beam.

Around the sample position, the environment is extremely crowded (Fig. 4 ▶), since, in addition to the gonio­meter system, the proper integration of the following units has to be ensured: the X-ray fluorescence detector (Ketek AXAS), the MS3 microspectrophotometer and its illumination unit objectives, the Robotic sample changer (IRELEC CATS; Ohana *et al.*, 2004 ▶), the Cryo-Shutter’s three-dimensional printed flexor design (Mueller *et al.*, 2012 ▶) (Fig. 5*a* ▶) and the cryo-cooling nozzle (CryojetXL by Oxford Instruments, Agilent Technologies). The nozzle needs to be retractable with full gas flow for quick intermittent room-temperature and plate-screening measurements and is opposed by a cold gas extraction tube (Fig. 5*b* ▶, bottom left).

The CATS sample changer is a wet mounting system, *i.e.* it grabs the sample pin in its vial, thereby keeping the sample immersed in liquid N_2_ during the transfer to the goniometer. It needs to access the goniometer along the direction of its rotation axis. This direction would also be optimal for two of the other devices: the Cryojet flow ideally should hit the pin at a shallow angle to minimize flow-induced vibrations. Further, since the synchrotron radiation is horizontally polarized, the background signal in the X-ray fluorescence spectra due to elastically scattered photons is minimal in the polarization direction (Dzubay *et al.*, 1974 ▶), so ideally the fluorescence detector should be positioned there as well. One solution to this integration problem would be a periscope-style detector finger that can easily be retracted from the path of the robot, for example like the system installed at the BESSY beamline 14.1 endstation (XFlash, Bruker AXS; Mueller *et al.*, 2012 ▶). With the PSI-standard Ketek AXAS systems, however, such a retractable positioning along the X-axis would be exceedingly bulky, and therefore a compromise was chosen, with a detector mounting from behind the goniometer at a 45° angle and a vertical pneumatic translation to park the detector. In a similar fashion, the Cryojet nozzle was oriented at an angle with respect to the X-axis to allow robotic mounting with minimal retraction of the nozzle to a distance to the sample of 12 mm during mounting and to 5 mm for alignment and data collection. A cryo-stream-extraction tube is installed opposite to the nozzle to immediately extract the cold gas and thereby prevent drifts due to cooling of other diffractometer parts. To avoid having to stop the cold flow of the cryo-system for intermittent room-temperature measurements, or for crystallization plate screening operation with the CATS system, the Cryojet nozzle can be retracted away from the sample position to an upstream parking position. Owing to a second cold gas extraction system there (currently in commissioning), the gas flow of the cryogenic system can be kept running, thereby avoiding long cool-down delays.

### 2.3. Beam shaping and diagnostics   

Micro-crystallography endstations employing pinholes or apertures as the last beam-defining element have previously been developed at the EMBL/ESRF (Perrakis *et al.*, 1999 ▶) and more recently, for example, at the GM/CA beamlines at the APS (Xu *et al.*, 2011 ▶), with a recent review listing current microcrystallography beamlines worldwide (Smith *et al.*, 2012 ▶). While they are not as flux-efficient compared with a beamline set-up where the full beam is refocused *via* a secondary focusing stage (Evans *et al.*, 2007 ▶), they are a good compromise for beamlines with sufficient photon flux to sacrifice some of it for the advantages of a microbeam.

The beam-shaping and diagnostics system (Fig. 5*b* ▶) is composed of three separate positioners, carrying (from upstream to downstream) a pinhole assembly, a scatter guard and collimator tube, and a combined scintillator/diode unit for beam diagnostics at the sample position. For the vertical translations, a positioning repeatability ≤1 µm was required, to ensure positioning to better than 10% of the size of the focused undulator beams. In addition to the high precision, the vertical positioners (Nanomotion HR2) also provide a travel range of 135 mm to retract for the CATS sample mounting and plate-screening grippers. The aperture and the collimator finger can also be scanned *via* horizontal positioners (SmarAct SLC-1720-S, travel range 8 mm), with an accuracy equal to the vertical translations. The scintillator finger can be scanned in the direction of the X-ray beam *via* a stack-type piezo-actuated flexor mechanism (travel range 0.5 mm) to bring it into the microscope focus.

**Figure 6:**
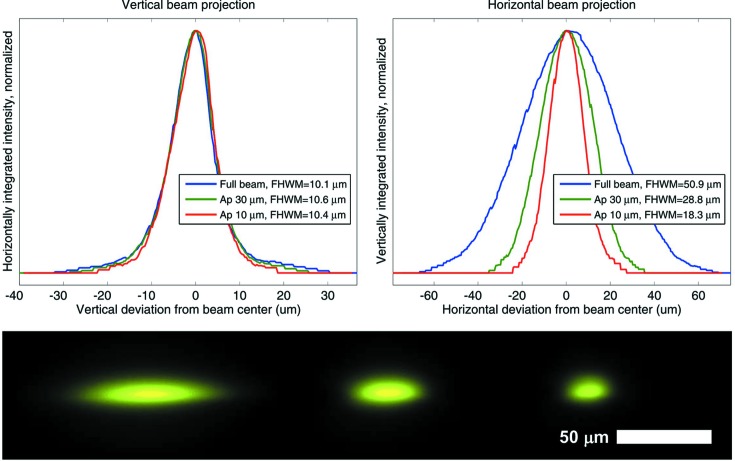
Top left: vertical beam projections obtained by integrating the intensity at the beam spot in the horizontal direction. Top right: horizontal beam projections. Bottom, from left to right: full beam, and beams through the 30 and 10 µm apertures, imaged by the on-axis sample microscope on a YAG:Ce scintillator screen on the third diagnostic device of the beam-shaping unit.

The aperture unit carries three vertically arranged pinholes developed for electron microscopy (Plano GmbH), 30 mm upstream of the sample position, with the drilled support acting as an upstream scatter guard. Currently three platinum apertures are available in sizes of 10, 30 and 150 µm with a thickness of 200 µm and an outer diameter of 3.04 mm. At beamline X10SA, with its unperturbed minimal focus size of 51 µm × 10 µm FWHM (H × V), the 10 µm aperture (Fig. 6 ▶) passes 10% and the 30 µm aperture passes 50% of the full flux of 2 × 10^12^ photons s^−1^ at 1 Å wavelength, which corresponds to a flux density of 2.5 × 10^9^ photons s^−1^ µm^−2^. This flux can be increased by another 75% to 3.5 × 10^12^ photons s^−1^ at 1 Å wavelength, by setting the gap of the U19 undulator to its minimum value of 4.5 mm. With the 10 µm aperture, a beam size of 18 µm × 10 µm can be achieved at the sample position, with the horizontal beam divergence leading to the larger horizontal beam size. By closing the tertiary slits in the beamline, approximately 0.4 m upstream of the sample, to lower the horizontal divergence at the expense of approximately 40% flux, a 10 µm × 10 µm size beam can be achieved. The 150 µm aperture is used either to pass the full beam or for measurements with defocused beams; for example, for large uniform crystals or for combined spectroscopy measurements to completely overlap the collinear spectroscopic beams. The collimator unit consists of a drilled 2.2 mm-diameter 2.5 mm-length brass scatter guard leading into a molybdenum tube with an outer diameter of 0.51 mm, an inner diameter of 0.30 mm and a length of 24 mm. Only the final 5 mm upstream of the sample are uncovered to avoid disturbing the N_2_ cryo gas stream.

Both the aperture and the collimator finger are connected to their positioners *via* a magnetic kinematic mount (Maag *et al.*, 2013 ▶), to allow for a quick and reproducible change to alternative aperture assemblies and also as a protection against accidental touching by operators or users. Directly underneath the magnetic mount, the positioner unit has two flexor tilt stages for rotational adjustment around the axes perpendicular to the beam.

The apertures are aligned to the beam by scanning them and recording the signal of the diode on the third diagnostic finger. Once the positions of the apertures have been optimized, typically once at the start of a user’s shift, the different apertures can be selected within seconds from the control GUI.

### 2.4. Beamstop   

**Figure 7:**
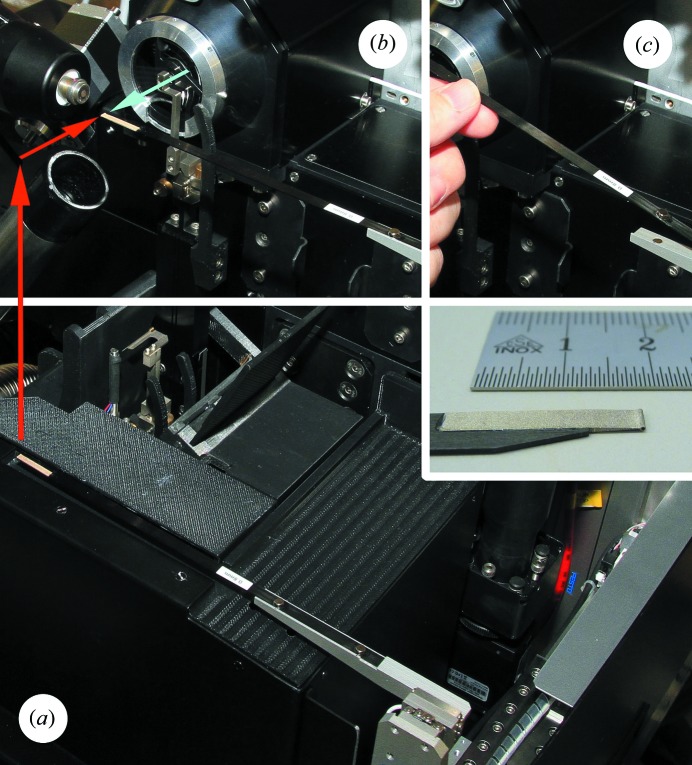
(*a*) Beamstop in the parked position, lowered (red arrow) with respect to the sample position (*b*) at the X-ray beam focus (tip of blue arrow). (*c*) Carbon blade fixation to beamstop mover *via* magnets and a reference bracket. Insert: wire-eroded Ag beamstop tip glued to a carbon blade.

The beamstop (Fig. 7 ▶) is fabricated from silver by wire erosion, with a thickness of 3 mm in the beam direction. The cylindrical portion hit by the X-ray beam has a 1 mm-deep hole to contain backscattered radiation, leaving 2 mm of material to absorb the beam. At beamline X10SA, with a photon energy range from 6 to 20 keV, two different outer diameters of 0.6 and 0.8 mm are provided, with the larger diameter used at photon energies above 16 keV to reliably suppress the powder pattern from the beam diffracted inside the beamstop.

Along the X-ray beam direction, the travel range is from −4 mm to 130 mm, with tilt degrees of freedom to allow for precise alignment of the linear stage along the beam. This offers users a choice of either minimizing the solid angle shadowed by the beamstop at the expense of a larger air-scattering background or *vice versa*. With a noise-free readout detector like the Pilatus 6M or 2M (Dectris) at the SLS MX beamlines, an optimized collection strategy can then consist of an initial low dose collection at a larger beamstop distance to obtain a high-quality complete low-order reflections dataset, followed by a second collection at a very short beamstop distance with accordingly lower background and a higher dose optimized for high-resolution data collection. Alternatively, to optimize the data acquisition for low-order reflections, at a wavelength of 2 Å with a 0.6 mm beamstop at 140 mm beamstop distance, theoretically diffraction orders to lower than 900 Å resolution become accessible. Practically, the current inner diameter of the collimator tube of 0.3 mm (see §2.3) in this case still lets pass a background halo of collimated secondary radiation around the beamstop. An upgraded collimator with a 0.1 mm bore end cap to block this background is currently being commissioned.

The beamstop is connected to a horizontal carbon arm and that in turn to the positioners that effect translations perpendicular to the beam (SmarAct SLC-1720-S, travel range ±8 mm). The carbon arm is held with magnets against a positioning bracket, which allows quick exchange and also prevents damage to both beamstop and positioners if this relatively exposed device is accidentally touched by operators. During sample mounting, the beamstop is retracted vertically into a groove in the diffractometer cover to further protect it.

For centering the beamstop on the beam, it is driven into the on-axis microscope focus at *Z* = 0 and then aligned visually in the sample-viewing GUI by simply clicking on the beamstop’s center.

### 2.5. Rapid alignment strategy   

The goniometer rotation axis position is usually aligned vertically with the GMY stage (Fig. 2 ▶) to intersect the X-ray beam at the start of a user shift by the support staff. While the beam position is generally very stable through energy changes, this stability relies on the proper calibration of the monochromator during commissioning and therefore can vary slightly. With the new diffractometer, the users can quickly relocate the beam position by choosing a beam location mode in the sample viewer and mark the beam focus at the sample on a scintillator image. From a possible deviation to the previous position, the required correctional movement of the goniometer translations is derived. In case the smallest aperture of 10 µm is used, a vertical beam movement by more than 2 µm also indicates a realignment of the pinhole, as described in §2.3.

### 2.6. Area detector   

A minimal detector distance of 120 mm can be achieved with the Pilatus 6M hybrid pixel area detector (Dectris). With a vertical detector height of 437 mm, at an X-ray wavelength of 1 Å this corresponds to a recordable resolution of 0.98 Å for reflections on the inscribed circle touching the vertical detector edge and to recordable resolutions of 0.61 Å and 2.02 Å at wavelengths of 0.62 Å (X-ray energy 20 keV) and 2.06 Å (6 keV), respectively. For the Pilatus 2M detector at beamline X06DA, we expect to reduce the minimal detector distance to below 90 mm due to the smaller detector size. With the vertical detector height of 289 mm, this shorter distance partly compensates for the smaller detector area, corresponding to a recordable resolution of 1.03 Å at the vertical detector edge at a wavelength of 1 Å.

### 2.7. Cover   

The diffractometer components close to the sample position and therefore most exposed to users’ interaction are covered by composite cover segments (see supporting information). The most complicated shape, the cover and lid of the beam-shaping devices unit, was manufactured by a 3D-printing rapid prototyping process (Stratasys Dimension Elite 3D printer with ABS material, PSI in-house fabrication). Together with the roof cover of the illumination unit, in the sample exchange state the cover forms a planar surface to optimally protect all components.

### 2.8. Table   

**Figure 8:**
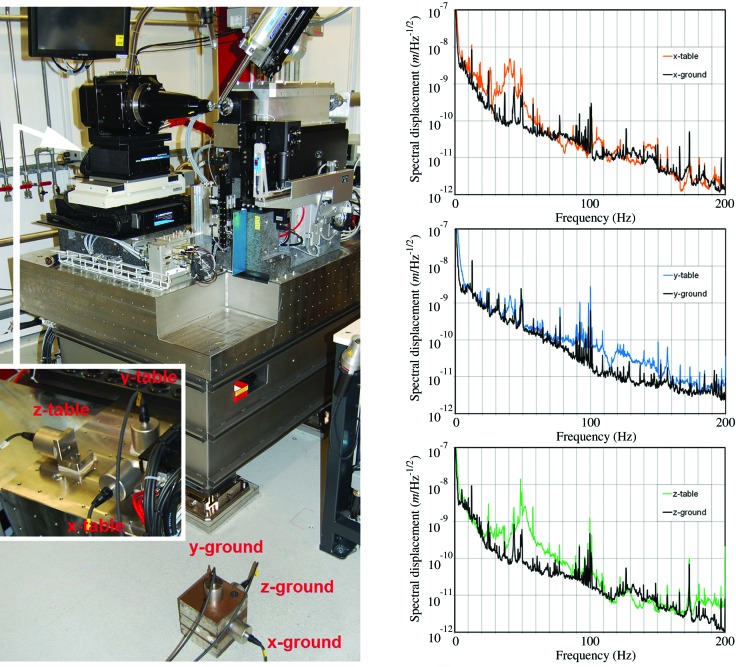
Left: measurement set-up for vibrations tests. Signals from accelerometers on the ground are compared with signals from accelerometers on the table (hidden behind the goniometer, see insert). The accelerometers were bolted horizontally *via* a rigid aluminium L-bracket, or glued vertically directly on the table. Right: comparison of ground vibrations (black line) and vibrations measured concurrently on the diffractometer table (colored line). *X* is transversal to the beam, *Y* is vertical and *Z* is along the beam. Plots of spectral displacement (square root of power spectral density of displacement, 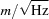), indicating the lowest vertical eigenfrequency of the table at 40 Hz. In the horizontal directions the width of the 40 Hz resonances extends to approximately 30 Hz.

For the diffractometer table (Fig. 8 ▶, left), a solution originally designed for the girders of the SwissFEL injector test facility (Wickström, 2010 ▶) was adapted. The main load-carrying structure is an epoxy-resin-bonded mineral cast block (material: EPUMENT 145/B, EPUCRET Mineral­gusstechnik). The block weighs approximately two tons and is contained in a stainless steel casting mold with a flat surface and a 50 mm × 50 mm breadboard thread pattern. The table block sits on three girder feet that provide translational adjustment and a tilt of the connecting interface. The table was constructed and the devices on top of it were aligned horizontally and then the whole assembly was tilted by 7 mrad to follow the X-ray beam inclination.

The most sensitive diffractometer components that do not extend down to the table surface, *i.e.* goniometer, beam-defining slits and the microspectrophotometer, are supported by separate granite blocks that are bolted to the table surface. This arrangement offers good stability and vibration damping while still enabling later reorganizations of components in case of a partial upgrade or re-design. The granite block supporting the goniometer stage was polished to a planarity of <5 µm as required by the flatness specifications of the bottom goniometer translation stage (GMX).

To quantify the vibration specifications of the diffractometer table construction, the power spectral density of the accelerations in all directions were measured concurrently on the hutch floor and on the table surface (Fig. 8 ▶, left). As specified in the design requirements for the undulator girder feet, the lowest main eigenfrequencies were observed at 40 Hz and the overall amplification factor was ≤1.8 (Fig. 8 ▶, right). The integrated displacements on the table from 5 to 200 Hz of measurement, obtained by double integration from the measured accelerations, were below 20 nm.

### 2.9. On-axis microspectrophotometer MS3   

**Figure 9:**
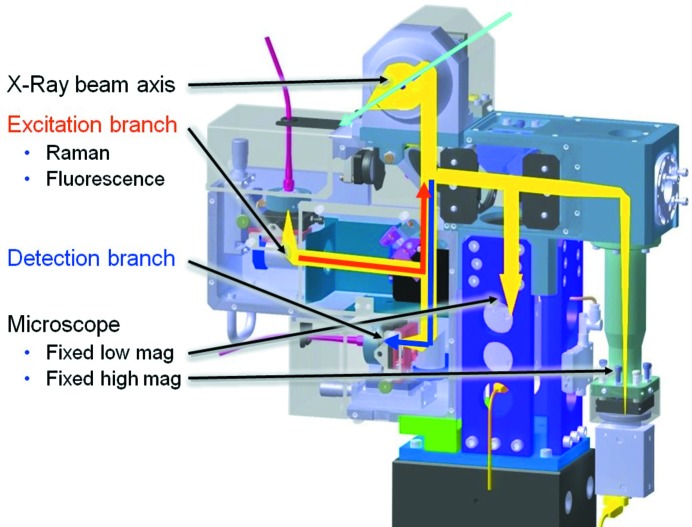
Beam paths of the MS3 microspectrophotometer. All optical axes converge at two beamsplitters underneath the reflective Schwarzschild microscope objective. From there they are deflected on-axis with the X-ray beam by a drilled 45° mirror behind the objective. The imaging branch of the sample-viewing microscope is split by a further beamsplitter into a low-magnification and a high-magnification fixed-zoom branch with separate firewire CCD cameras (Point Grey GRAS-20S4-C). The spectroscopic unit contains the beamsplitter separating the excitation from the detection branch, both coupled *via* light fibers to various lasers and the spectrographs, respectively.

**Figure 10:**
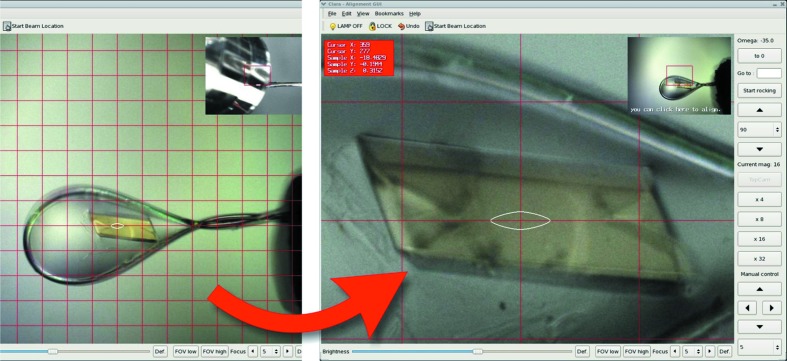
MS3 microscope image. The two concurrent fixed zoom levels enable the display of a picture-in-picture image. The loop can thus be aligned in the small low-magnification view and the crystal subsequently in the high-magnification main view without having to change zoom levels.

**Figure 11:**
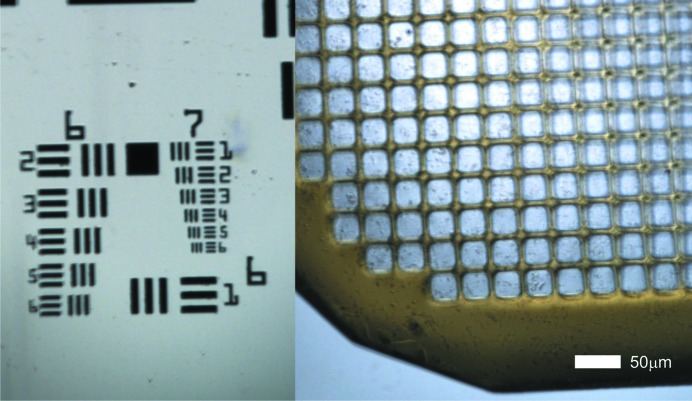
MS3 microscope image resolution. Left: image of a 1951 USAF resolution test pattern conforming to MIL-STD-150A standard. The smallest three-line-pattern, Group 7/Element 6, is still well resolved. It corresponds to a resolution of 228 line pairs per mm, *i.e.* the width of one line is 2.19 µm. Right: image of a Mitegen MicroMesh (700/25) carrying a suspension of microcrystals of virus spheroids with an average crystal size of 1–2 µm.

One of the design goals of the new D3 diffractometer was the inclusion of a microspectrophotometer into the on-axis viewing system (Fig. 9 ▶). The basis of the adopted design is the SLS Microspectrophotometer version 2 (MS2) (Pompidor *et al.*, 2013 ▶), with modifications to the sample-viewing branches to support two concurrent views (Fig. 10 ▶) with fixed magnifications of 2.8 and 13 (on-camera) and an image resolution of 1 µm in the high-magnification view (Fig. 11 ▶). The fields of view are 1.6 mm × 1.2 mm and 0.35 mm × 0.26 mm, respectively.

With the new device, both the microscope and the spectrometer functionalities are always online, providing the possibility to perform X-ray diffraction measurements with concurrent UV/Vis, fluorescence and Raman and resonance Raman spectroscopic experiments. Setting up the spectroscopic modes requires a preparation time of typically less than 30 min and the switching time between the modes is just a few minutes without the need to remove the sample from the goniometer during the change.

Several optical figures of merit (FOM) were considered in the design of the microscope: image resolution, spectral bandwidth, depth of field, working distance and general ‘image quality’-related parameters. The required image resolution of 1 µm, as well as the requirement to sample a large solid angle for spectroscopy, guides the choice of a large numerical aperture, and thereby the width of the optical components and the size of the complete unit. The spectral bandwidth of reflective objectives is considerably higher in the UV range and therefore indicates their use for the design of a microspectrophotometer. A drawback of a reflective objective design is that the central obscuration limits the use of an iris to reduce the numerical aperture, and thereby the possibility to maximize the depth of field. The choice of working distance, 35 mm, was guided by the requirement to be able to integrate more space-demanding methods such as multi-axis goniometry and *in situ* plate screening. All other parameters influencing the perceived ‘image quality’, such as contrast, dynamic range or stray light background, can be optimized by the choice of system components without compromises between different FOMs. The microscope objective for beamline X10SA was optimized with the spectroscopy applications in mind, and therefore a reflective Schwarzschild objective based design was implemented. The rapid and reproducible focusing mechanism based on the goniometer translation along the beam allows for easy compensation of the limitation in field depth for large samples.

**Figure 12:**
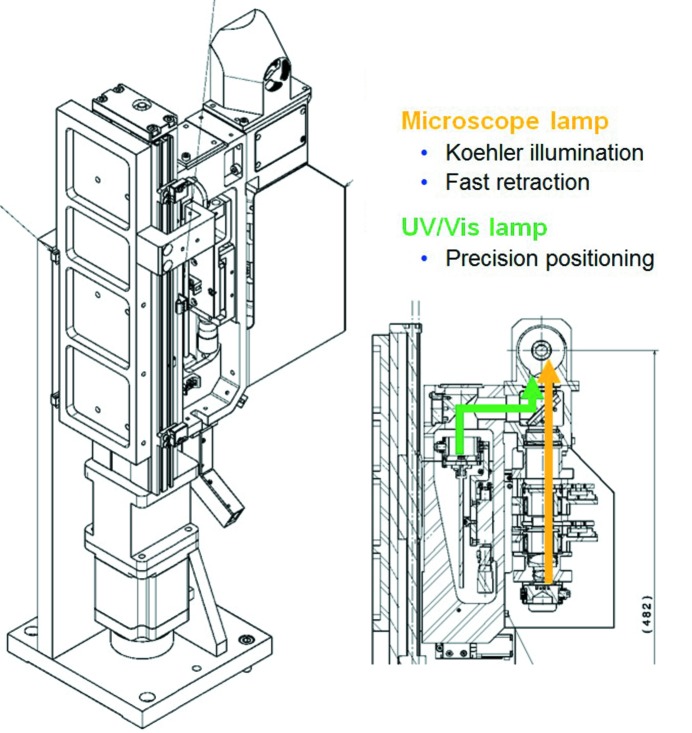
Left: ilumination unit of the microspectrophotometer MS3. The unit is a combination of a Köhler-type microscope light and a light guide coupled to an absorption spectroscopy broadband white-light source. A 190 mm vertical translation with a 1 µm resolution permits the unit to be retracted within 2 s. Lateral positioning is provided by the vertical translation and a second horizontal stage (range 10 mm, resolution 1.25 µm). Right: beam paths in the MS3 illumination unit. The vertical beam path of the Köhler illumination comprises (from bottom to top) an LED light source, two lens doublets, an iris to define the illuminated area, a polarizer, a third doublet and a reflective objective. The beam path of the spectroscopic illumination contains the fiber holder, an off-axis parabolic mirror and a beam splitter to couple the light into the reflective objective along with the Köhler illumination branch.

The illumination unit (Fig. 12 ▶) combines the functions of a microscope back light and of an absorption spectroscopy white-light illumination objective. The two use cases lead to very different requirements for the motorized vertical translation of the unit: for the sample-viewing function, the rapid raising and lowering of the unit is of high importance to minimize wait times during the experiment state changes. For the spectroscopic illumination, the beam size is matched to the typical crystal sizes and therefore requires positioning with micrometer precision. To accommodate the two requirements of high speed and high precision, the stepper motor is actuated at a very high pulse rate of 100 kHz.

The detailed optical layout of the spectroscopy branches of the MS3 as well as the off-line SLSpectroLab will be described in a separate article.

### 2.10. Cryo-stream extraction   

Temperature stability is essential for the long-term stability of the diffractometer configuration, particularly when the smallest beam-defining apertures are used. To keep the N_2_ cooling gas from selectively cooling parts of the diffractometer and thereby inducing drifts, a cryo-gas extraction tube was installed (Fig. 5*b* ▶, bottom left). By choosing a larger diameter for the extraction tube compared with the Cryojet cooling nozzle, the cold gas is mixed with room-temperature air, which keeps the tubes from accumulating condensed air humidity.

In addition, the prototype of a more narrow and retractable extraction tube is being evaluated, which provides sufficient working space for sample exchange but can be brought in close proximity to the sample during measurements. Such an extraction tube in close proximity to the Cryojet nozzle leads to a more laminar flow and therefore is expected to minimize vibrations of the sample, with a possible improvement in data quality (Alkire *et al.*, 2008 ▶, 2013 ▶). As for this configuration, relatively more cold gas enters the extraction tube; a Macor tube with a heating coil is used to prevent ice formation on the tube.

## 3. Controls software   

Care was taken to keep the diffractometer compatible with the established control system based on EPICS and a client-server-based GUI structure. Since August 2012, the new controls GUI for the SLS MX beamlines, DA+, based on a Java Eclipse RCP GUI, has been put in general user operation. The detailed design of the software will be published separately.

The goniometer is controlled by an EPICS IOC running on a Microsoft Windows PC, interfaced *via* Firewire to an Aerotech Automation A3200 controller with the Motion Composer controls suite (software version 4.x). The Aerotech controller is programmed using the Aerobasic language to implement synchronized movements and position synchronized outputs (PSO) set on specified encoder positions on the various axes controlled by the system.

The general experiment control is organized by an Experiment State Controller tool called ESCAPE, with the main experiment states being ‘Sample Exchange’, ‘Sample Alignment’ and ‘Data Collection’. Each transition from one state to another is carried out in the most efficient and safe way, thus avoiding hardware collisions. ESCAPE also allows for different ‘models’, with each model having specific motions to carry out the change between states. Currently implemented ESCAPE models include ‘Manual’, ‘Sample Changer’, ‘No Movements’ and ‘Spectroscopy’. Different models can be easily added to accommodate user-specific hardware mounted at the endstation. Within the states, movement of all devices required for the respective experimental task is then possible from the control GUI within collision-free travel ranges. For free movement of all devices, an additional ‘Maintenance’ state is available.

In contrast to the previous diffractometer set-up, the spectroscopy operation does not have any implications on the number of movable devices, and therefore is covered by the standard experiment states.

Almost all of the additional controls functionality required for the commissioning of the instrument during the set-up for user operation were brought together in a single EPICS MEDM panel, the ‘X10SA ONE’ panel. This panel already greatly facilitates beamline set-up and commissioning; eventually it will be integrated with the general DA+ GUI as an expert feature.

## 4. Conclusion   

The first D3 diffractometer has been in continuous user operation at beamline X10SA since April 2012. The next instance of the device is currently being installed at beamline X06SA alongside a two-stage microfocus upgrade of the beamline optics. As the microfocus upgrade was initiated after the finalization of the original diffractometer design, the next version of the D3 will be implemented with some modifications, *e.g.* to accommodate the vacuum tank of the secondary focusing stage. The versatility of the blocks-on-table approach adopted for the basic diffractometer layout has already proved crucially important in implementing the required changes, which will be published along with the microfocusing upgrade. The upgrade of the diffractometer of the youngest SLS MX beamline, X06DA, will conclude the rollout.

The improvements in the diffractometer performance and its ease of use have been very well received by the user community. With the beam-shaping unit, users can rapidly re-shape the beam size to more closely match their sample size, and quickly verify its position and flux. Moreover, the new small beams, along with the small cylinder of confusion of the goniometer, have enabled microcrystallography measurements. Combined with the precision piezo-centering stages and the updated controls software, accurate rastering for tiny crystals and mapping the diffraction quality of large crystals are offered to the users, as well as advanced data acquisition schemes like helical scanning. All spectroscopic modes of the microspectrophotometer are now available without the need to rebuild the set-up. Moreover, the time required for the set-up and maintenance of the instrument has been greatly reduced, thereby facilitating user support and maintenance work. Once all three beamlines have been upgraded, any further instrumentation developments or improvements on one of them can directly be carried over to all beamlines. The new endstation design, providing state-of-the-art performance at the time of installation, with its openness to extensions will therefore continue to be a reliable basis for future developments and upgrades for the SLS MX endstations.

## Supplementary Material

Supplementary Fig. 1: Goniometer metrology setup for cylinder of confusion commissioning.. DOI: 10.1107/S160057751400006X/rv5010sup1.pdf

Supplementary Fig. 2: Sample environment with the Pilatus 6M detector. DOI: 10.1107/S160057751400006X/rv5010sup2.pdf

Supplementary Fig. 3: Diffractometer cover close to sample.. DOI: 10.1107/S160057751400006X/rv5010sup3.pdf

## Footnotes

1 Supporting information for this paper is available from the IUCr electronic archives (Reference: RV5010).

## References

[bb1] Aishima, J., Owen, R. L., Axford, D., Shepherd, E., Winter, G., Levik, K., Gibbons, P., Ashton, A. & Evans, G. (2010). *Acta Cryst.* D**66**, 1032–1035. 10.1107/S0907444910028192 PMC6691516 20823554

[bb2] Alkire, R. W., Duke, N. E. C. & Rotella, F. J. (2008). *J. Appl. Cryst.* **41**, 1122–1133. 10.1107/S1600576716000431 PMC4815871 27047303

[bb3] Alkire, R. W., Rotella, F. J. & Duke, N. E. C. (2013). *J. Appl. Cryst.* **46**, 525–536. 10.1107/S1600576716000431 PMC4815871 27047303

[bb4] Ascone, I., Girard, E., Gourhant, P., Legrand, P., Roudenko, O., Roussier, L. & Thompson, A. W. (2007). *AIP Conf. Proc.* **882**, 872–874.

[bb5] Bingel-Erlenmeyer, R., Olieric, V., Grimshaw, J. P. A., Gabadinho, J., Wang, X., Ebner, S. G., Isenegger, A., Schneider, R., Schneider, J., Glettig, W., Pradervand, C., Panepucci, E. H., Tomizaki, T., Wang, M. & Schulze-Briese, C. (2011). *Cryst. Growth Des.* **11**, 916–923.

[bb6] BioSync (2013). *Biosync: A Structural Biologist’s Guide to High Energy Data Collection Facilities*, http://biosync.sbkb.org.

[bb7] Cherezov, V., Hanson, M. A., Griffith, M. T., Hilgart, M. C., Sanishvili, R., Nagarajan, V., Stepanov, S., Fischetti, R. F., Kuhn, P. & Stevens, R. C. (2009). *J. R. Soc. Interface*, **6**, S587–S597. 10.1098/rsif.2009.0142.focus PMC2843980 19535414

[bb8] Diez, J., Wang, M., Pohl, E., Tomizaki, T., Bertrand, A., Chen, Q., Dietrich, P., Ingold, G., Knecht, M., Meents, A., Olieric, V., Panepucci, E., Pauluhn, A., Pradervand, C., Roccamante, M., Schneider, R., Walthert, I., Zimoch, E. & Schulze-Briese, C. (2007). *Synchrotron Radiat. News*, **20**, 19–22.

[bb9] Donaldson, R. R. (1972). *CIRP Ann.* **21**, 125–126.

[bb10] Dzubay, T., Jarrett, B. & Jaklevic, J. (1974). *Nucl. Instrum. Methods*, **115**, 297–299.

[bb11] EPICS (2012). *The Experimental Physics and Industrial Control System*, http://www.aps.anl.gov/epics/.

[bb12] Evans, G., Alianelli, L., Burt, M., Wagner, A. & Sawhney, K. J. S. (2007). *AIP Conf. Proc.* **879**, 836–839.

[bb13] Fischer, P., Reime, B., Stuebe, N., Pakendorf, T. & Meents, A. (2012). *Acta Cryst.* A**68**, s268.

[bb14] Flot, D., Mairs, T., Giraud, T., Guijarro, M., Lesourd, M., Rey, V., van Brussel, D., Morawe, C., Borel, C., Hignette, O., Chavanne, J., Nurizzo, D., McSweeney, S. & Mitchell, E. (2010). *J. Synchrotron Rad.* **17**, 107–118. 10.1107/S0909049509041168 PMC3025444 20029119

[bb15] Glettig, W., Vitins, M., Schwarb, A., Maag, S. & Schulze-Briese, C. (2011). *Proceedings of the 11th Euspen International Conference*, pp. 31–34. Como: Euspen.

[bb16] Goulet, A., Vestergaard, G., Felisberto-Rodrigues, C., Campanacci, V., Garrett, R. A., Cambillau, C. & Ortiz-Lombardía, M. (2010). *Acta Cryst.* D**66**, 304–308. 10.1107/S0907444909051798 20179342

[bb17] Maag, S., Frommherz, U., Kotrle, G., Thominet, V., Welte, J., Pradervand, C., Thermer, R., Schnorf, D., Ellenberger, U. & Fuchs, M. R. (2013). *J. Phys. Conf. Ser.* **425**, 012009.

[bb18] Mueller, U., Darowski, N., Fuchs, M. R., Förster, R., Hellmig, M., Paithankar, K. S., Pühringer, S., Steffien, M., Zocher, G. & Weiss, M. S. (2012). *J. Synchrotron Rad.* **19**, 442–449. 10.1107/S0909049512006395 PMC3408958 22514183

[bb19] Ohana, J., Jacquamet, L., Joly, J., Bertoni, A., Taunier, P., Michel, L., Charrault, P., Pirocchi, M., Carpentier, P., Borel, F., Kahn, R. & Ferrer, J.-L. (2004). *J. Appl. Cryst.* **37**, 72–77.

[bb20] Perrakis, A., Cipriani, F., Castagna, J.-C., Claustre, L., Burghammer, M., Riekel, C. & Cusack, S. (1999). *Acta Cryst.* D**55**, 1765–1770. 10.1107/s0907444999009348 10531527

[bb21] Pohl, E., Pradervand, C., Schneider, R., Tomizaki, T., Pauluhn, A., Chen, Q., Ingold, G., Zimoch, E. & Schulze-Briese, C. (2006). *Synchrotron Radiat. News*, **19**, 24–26.

[bb22] Pompidor, G., Dworkowski, F. S. N., Thominet, V., Schulze-Briese, C. & Fuchs, M. R. (2013). *J. Synchrotron Rad.* **20**, 765–776. 10.1107/S0909049513016063 PMC3747950 23955041

[bb23] Rosenbaum, G., Alkire, R. W., Evans, G., Rotella, F. J., Lazarski, K., Zhang, R.-G., Ginell, S. L., Duke, N., Naday, I., Lazarz, J., Molitsky, M. J., Keefe, L., Gonczy, J., Rock, L., Sanishvili, R., Walsh, M. A., Westbrook, E. & Joachimiak, A. (2006). *J. Synchrotron Rad.* **13**, 30–45. 10.1107/S0909049505036721 PMC2603069 16371706

[bb24] Salathe, M. (2010). Internship Report. Paul Scherrer Institute and Ecole Polytechnique Federale de Lausanne, Switzerland.

[bb25] Schulze-Briese, C., Tomizaki, T., Pradervand, C., Schneider, R., Janousch, M., Portmann, W., Chen, Q., Ingold, G., Rossetti, D., Frauenfelder, B., Zumbach, C., Hottinger, P., Brönnimann, C. & Eikenberry, E. F. (2001). *Scientific Report 2007*, Vol. VII, pp. 54–55. Paul Scherrer Institute, Villigen PSI, Switzerland.

[bb26] Smith, J. L., Fischetti, R. F. & Yamamoto, M. (2012). *Curr. Opin. Struct. Biol.* **22**, 602–612. 10.1016/j.sbi.2012.09.001 PMC3478446 23021872

[bb27] Song, J., Mathew, D., Jacob, S. A., Corbett, L., Moorhead, P. & Soltis, S. M. (2007). *J. Synchrotron Rad.* **14**, 191–195. 10.1107/S0909049507004803 17317920

[bb28] Wickström, J. (2010). *SwissFEL Injector Conceptual Design Report*, p. 29. Paul Scherrer Institute, Villigen PSI, Switzerland.

[bb29] Xu, S., Makarov, O., Benn, R., Yoder, D. W., Stepanov, S., Becker, M., Corcoran, S., Hilgart, M., Nagarajan, V., Ogata, C. M., Pothineni, S., Sanishvili, R., Smith, J. L. & Fischetti, R. F. (2010). *AIP Conf. Proc.* **1234**, 905–908.

[bb30] Xu, S., Nagarajan, V. & Fischetti, R. F. (2011). *Diamond Light Source Proc.* p. e27.

