# Supplementary material for: D3, the new diffractometer for the macromolecular crystallography beamlines of the Swiss Light Source
Source: J Synchrotron Radiat. 2014 Feb 4;21(Pt 2):340–51. doi: 10.1107/S160057751400006X (PMC3945418; doi:10.1107/S160057751400006X)
Supplement: Supplementary file 1 [file s-21-00340-sup1.pdf]

Supplementary material

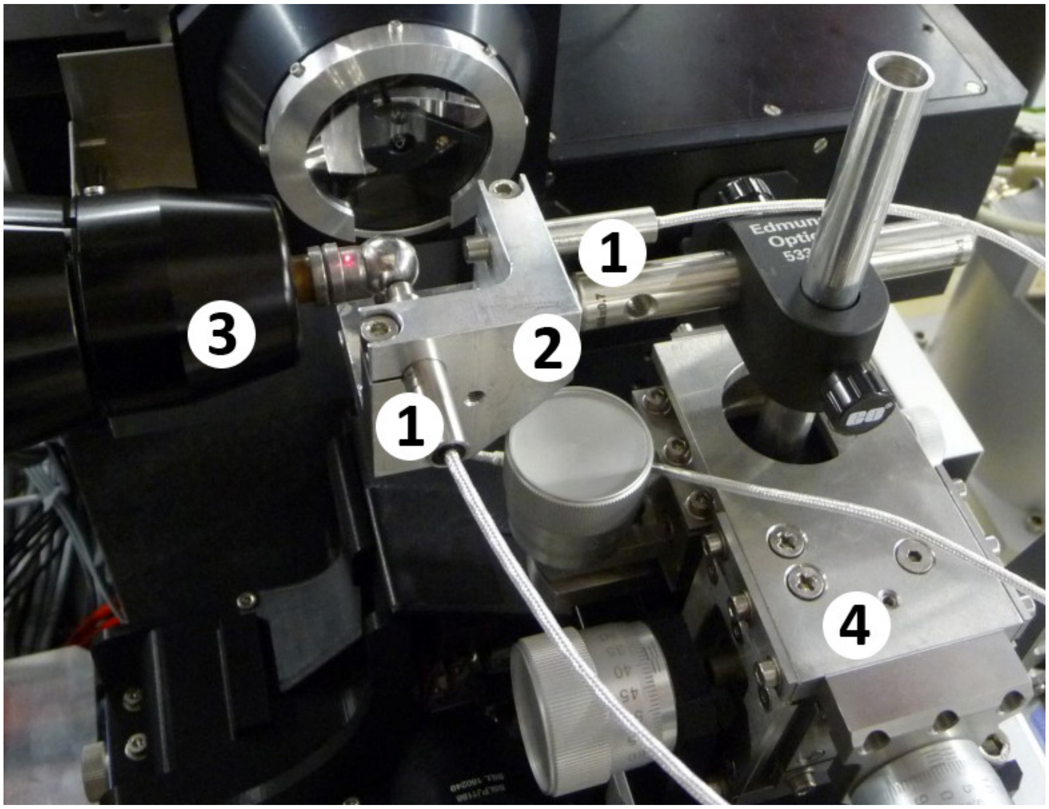

Figure 1 Goniometer metrology setup for cylinder of confusion commissioning. Three capacitive sensors (1) are mounted in an easy-to-adjust bracket (2), one for each spatial direction. The sensors measure the distance to the reference sphere at the sample position. The sphere is centered by the goniometer's centering stages (3) and the capacitive sensors are manually adjusted by an XYZ stage (4).
