# Supplementary material for: D3, the new diffractometer for the macromolecular crystallography beamlines of the Swiss Light Source
Source: J Synchrotron Radiat. 2014 Feb 4;21(Pt 2):340–51. doi: 10.1107/S160057751400006X (PMC3945418; doi:10.1107/S160057751400006X)
Supplement: Supplementary file 2 [file s-21-00340-sup2.pdf]

Supplementary material

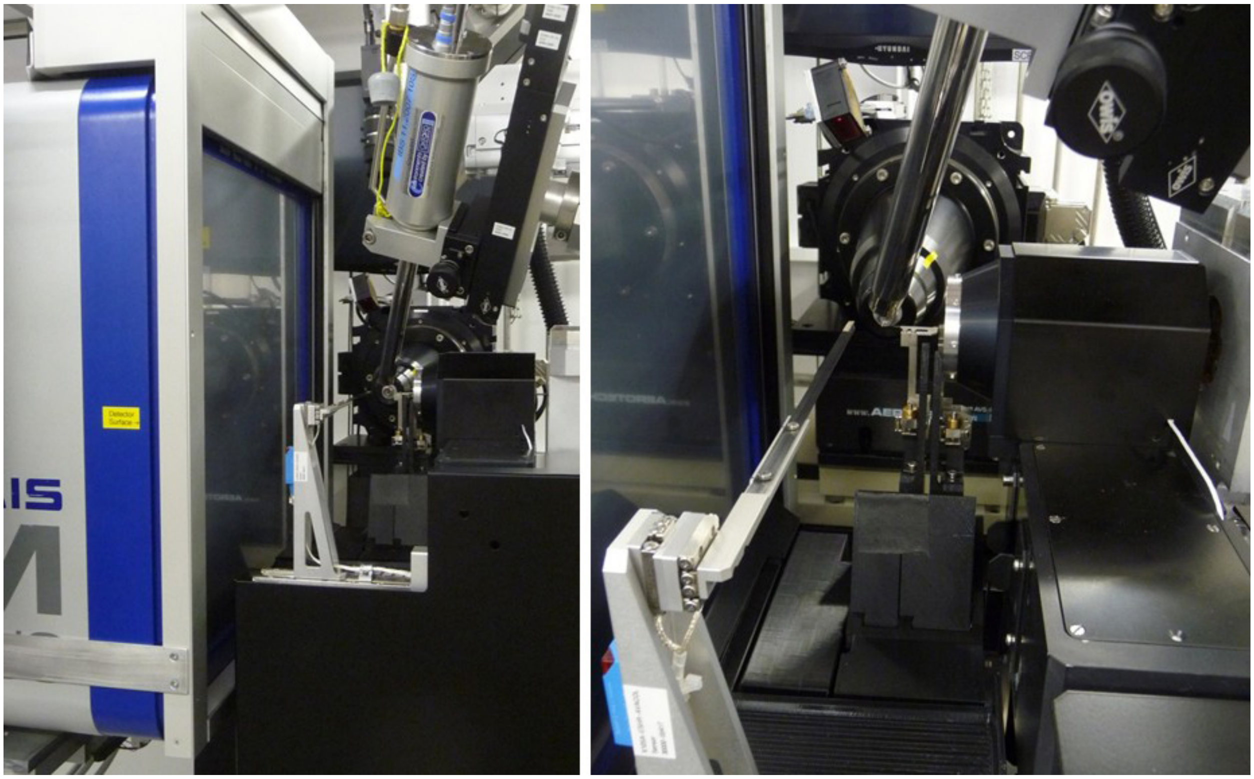

Figure 2 Left: Sample environment with the Pilatus 6M detector with opened protective cover. Right: Sample environment in Data Acquisition state, with retracted illumination and extended beam shaping devices.
