# Supplementary material for: D3, the new diffractometer for the macromolecular crystallography beamlines of the Swiss Light Source
Source: J Synchrotron Radiat. 2014 Feb 4;21(Pt 2):340–51. doi: 10.1107/S160057751400006X (PMC3945418; doi:10.1107/S160057751400006X)
Supplement: Supplementary file 3 [file s-21-00340-sup3.pdf]

Supplementary material

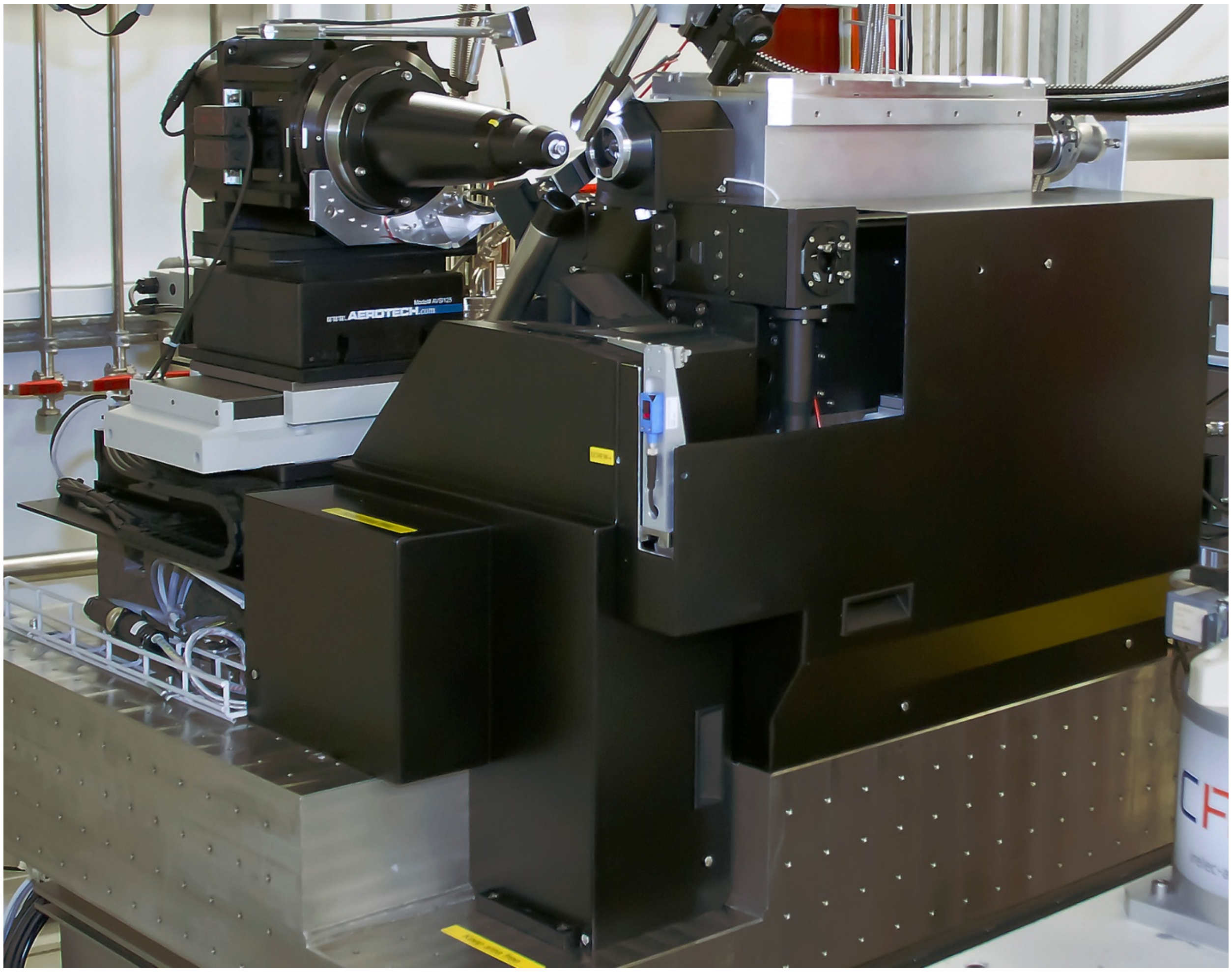

Figure 3 Diffractometer cover close to sample. A two-piece Al-cover protects all cabling and actuators of the diffractometer with the exception of the goniometer at left.
